# Rheumatoid arthritis-associated gene-gene interaction network for rheumatoid arthritis candidate genes

**DOI:** 10.1186/1753-6561-3-s7-s75

**Published:** 2009-12-15

**Authors:** Chien-Hsun Huang, Lei Cong, Jun Xie, Bo Qiao, Shaw-Hwa Lo, Tian Zheng

**Affiliations:** 1 Department of Statistics, Columbia University, 1255 Amsterdam Avenue, 10th Floor, MC44690, New York, New York 10027, USA; 2 Department of Statistics, 250 North University Street, Purdue University, West Lafayette, Indiana 47907, USA

## Abstract

Rheumatoid arthritis (RA, MIM 180300) is a chronic and complex autoimmune disease. Using the North American Rheumatoid Arthritis Consortium (NARAC) data set provided in Genetic Analysis Workshop 16 (GAW16), we used the genotype-trait distortion (GTD) scores and proposed analysis procedures to capture the gene-gene interaction effects of multiple susceptibility gene regions on RA. In this paper, we focused on 27 RA candidate gene regions (531 SNPs) based on a literature search. Statistical significance was evaluated using 1000 permutations. *HLADRB1 *was found to have strong marginal association with RA. We identified 14 significant interactions (*p *< 0.01), which were aggregated into an association network among 12 selected candidate genes *PADI4*, *FCGR3*, *TNFRSF1B*, *ITGAV*, *BTLA*, *SLC22A4*, *IL3*, *VEGF*, *TNF*, *NFKBIL1*, *TRAF1-C5*, and *MIF*. Based on our and other contributors' findings during the GAW16 conference, we further studied 24 candidate regions with 336 SNPs. We found 23 significant interactions (*p*-value < 0.01), nine interactions in addition to our initial findings, and the association network was extended to include candidate genes *HLA-A*, *HLA-B*, *HLA-C*, *CTLA4*, and *IL6*. As we will discuss in this paper, the reported possible interactions between genes may suggest potential biological activities of RA.

## Background

Rheumatoid arthritis (RA) is a common inflammatory disorder with complex etiology. Although the causes of RA are still unclear, it is believed to be attributed to both genetic and environmental factors. During the last few decades, many new genetic regions have been identified to associate with RA. Remmers et al. [1] showed that *STAT4 *was an important genetic marker for both RA and systemic lupus erythematosus susceptibility. Kurreeman et al. [2] applied the candidate gene approach to the *TRAF1-C5 *region and found a polymorphism that increased the susceptibility and severity of RA. Plenge et al. [3] studied 14 candidate genes and found significant associations between RA and *PTPN22*, *CTLA4*, and *PADI4*. Although many genes have shown suggestive connections with RA, only *HLADRB1 *and *PTPN22 *have been confirmed to increase the genetic risk of developing RA [4]. An explanation for the large number of identified genes might lie in the existence of gene-gene interactions, which, while helpful in identifying and compiling genes, also made the analysis much more complicated.

The traditional approaches used in association studies analyze markers marginally one at a time. As a result, valuable information on the interactions of genes was lost. Zheng et al. [5] proposed an association measure, the genotype-trait distortion (GTD), for evaluating association information on unphased multilocus genotypes from case-control data. GTD was shown to be able to capture interactions between markers that were associated with the disease [5].

Many current association studies are based on dense single-nucleotide polymorphism (SNP) data, with multiple SNPs corresponding to one gene. SNP-based methods are used to identify and replicate the most significant SNP. Without considering the dependence and functional relevance among SNPs within the same gene, most SNP-based association analysis may lead to false-negative results. As marker density increases, one could consider a gene-based analysis that offers a number of advantages, for instance, taking into account possible multiple disease-associated functional variants within a gene and overcoming the dependence among SNPs due to close proximity. In this study, we used the GTD score and applied a gene-based analysis on two sets of RA candidate genes from the North American Rheumatoid Arthritis Consortium (NARAC) data as part of Problem 1 of the Genetic Analysis Workshop 16 (GAW16).

## Methods

### Gene Set I

The Illumina data set of GAW16 Problem 1 consists of 545,080 SNPs genotyped on 868 RA cases and 1194 unaffected controls from NARAC. In our study, 531 SNPs from 27 candidate gene regions were considered (Table 1). The SNPs numbers and locations were identified by using the SNP mapping information from the National Cancer Institute's Cancer Genetic Markers of Susceptibility (CGEMS) initiative [6]. We included all related SNPs within each gene in the analysis. These 27 candidate genes were selected based on our search of the RA literatures. Among these 27 candidate genes, *HLADRB1 *and *PTPN22 *have been widely studied and confirmed to be associated with RA risk. Some of the candidate genes (*PADI4*, *FCRL3*, *FCGR3*, *TNFRSF1B*, *STAT4*, *CTLA4*, *IL4*, *HAVCR1*, *TNF*, *MICA*, *NFKBIL1*, *OLIG3*-*TNFAIP3*, *TRAF1*-*C5*, *MHC2TA*, and *MIF*) have been replicated in different populations. A few candidate genes (*IL10*, *BTLA*, *IL1B*, *ITGAV*, *SLC22A4*, *IL3*, *VEGF*, and *RUNX1*) showed positive association in individual studies but have not been replicated in other independent data sets. In addition, we also included *DLG5 *and *CARD15 *because they were found to be associated with several autoimmune diseases. SNPs with missing data were imputed by fastPhase [7].

**Table 1:** RA candidate genes

Gene	Gene Set I	Gene Set II	Locus	SNPs
*PTPN22*	Yes	Yes	1p13	12
*PADI4*	Yes	Yes	1p36.13	18
*FCRL3*	Yes	No	1q21.2-q22	8
*FCGR3*	Yes	Yes	1q23	4
*IL10*	Yes	No	1q31-q32	16
*TNFRSF1B*	Yes	Yes	1p36.3-p36.2	21
*IL1B*	Yes	Yes	2q14	7
*ITGAV*	Yes	Yes	2q31	16
*STAT4*	Yes	Yes	2q32.2-q32.3	30
*CTLA4*	Yes	Yes	2q33	5
*BTLA*	Yes	Yes	3q13.2	6
*SLC22A4*	Yes	Yes	5q31	19
*IL13*	No	Yes	5q31	6
*IL3*	Yes	Yes	5q31.1	3
*IL4*	Yes	No	5q31.1	7
*HAVCR1*	Yes	No	5q33.2	15
*VEGF*	Yes	Yes	6p12	12
*HLADRB1*	Yes	Yes	6p21.3	5
*MICA*	Yes	No	6p21.3	32
*HLA-A*	No	Yes	6p21.3	6
*HLA-B*	No	Yes	6p21.3	24
*HLA-C*	No	Yes	6p21.3	29
*LTA*	No	Yes	6p21.3	11
*NFKBIL1*	Yes	Yes	6p21.3	19
*TNF*	Yes	Yes	6p21.3	10
*OLIG3 - TNFAIP3*	Yes	No	6q23	76
*IL6*	No	Yes	7q21	14
*TRAF1-C5*	Yes	Yes	9q33-q34	31
*DLG5*	Yes	No	10q23	17
*MS4A1*	No	Yes	11q31	13
*MHC2TA*	Yes	No	16p13	12
*CARD15*	Yes	No	16q12	11
*RUNX1*	Yes	No	21q22.3	104
*MIF*	Yes	Yes	22q11.2	15

### Gene Set II

After the gene × gene interaction group discussion during the GAW16 conference, 24 candidate genes were selected for further analysis based on the contributors' findings (Table 1), 17 of which were considered in our original analysis (Gene Set I). The seven added genes are *IL13*, *HLA-A*, *HLA-B*, *HLA-C*, *LTA*, *IL6*, and *MS4A1*, which were identified in other contributors' studies. In other words, we applied our method to re-analyze the same RA data with different candidate genes from Gene Set I.

## Methods

Given *k *SNP makers, there are 3^*k *^possible unphased genotypes. We can use the following GTD statistic defined on the sum of squared difference between genotypes' relative frequency among the cases and controls to measure the joint effects of these *k *SNPs on the disease status, i.e.,

The statistic *ν *and its variations have been applied successfully in a number of studies [5,8-10]. Specifically, GTD was applied to a candidate gene study of RA during GAW15 [10] and identified significant higher-order interactions that were missed by other methods such as the multifactor dimensionality reduction (MDR) [10-12].

To identify significant interactions, we applied 1000 permutations of the case-control outcomes as in Lo et al. [13]. Based on *ν*, we applied the following gene-based analysis procedures developed by Lo et al. [13] to analyze our data sets:

1. Suppose that there are *l *genes with *m*_*i *_SNPs in gene *i *and the total number of SNPs is *m *(). For example, *l *= 27 and *m *= 531 in Gene Set I. We calculated  for SNP *d *of gene *i *(*k *= 1) and took an average over the *m*_*i *_SNPs for each gene *i*, which resulted in *l *averages of the marginal effect of each gene.

2. We randomly permuted the labels of case (*Y *= 1) and control (*Y *= 0) and repeated Step 1 on the permuted data while the original dependence among SNPs in each gene were retained.

3. To find the pair-wise interaction among the *l *genes, we examined *l *× (*l*-1)/2 gene pairs. We first calculated the interaction genotype statistics  for *m *× (*m*-1)/2 SNP pairs (*k *= 2) and defined the SNP-wise interaction as the ratio of incremental interaction versus the maximum of the two marginal effects as in Eq. (2) where "∨ " stands for maximum of the two values.

Second, we defined the interaction between genes *i *and *j *as the average of all SNP-wise ratios in Eq. (3). The ratio is termed as the "mean interaction ratio," *Mean-ratio*, or *R *statistic. In addition, we also defined the gene-level "average maximum marginal," or *M*, as in Eq. (4).

4. Applying the calculation outlined in Step 3 on the permuted data sets, we obtained a set of *l *× (*l*-1)/2 values {} for each permutation *p*. The *Mean-ratio curve method *of the following was applied to identify significant gene-pairs. All 1000 × *l *× (*l*-1)/2 points were put on the (*M, R*) plane and separated into 100 bins according to the *M *values. Each bin had 10 × *l *× (*l*-1)/2 points. For each bin, we identified the 99 percentile of the *R *statistics (R*) that fell within this bin. By fitting a smoothing spline between the mid-values of each bin (M*) and R*, we constructed the 99% *R *threshold curve conditioning on *M*. We identified gene-pairs with observed *R *statistics above the threshold curve as significant.

5. In addition to the mean ratio *R*, we used another measure of interaction, the *Quantile-ratio *statistic. The *Q *statistic is calculated as the 90^th ^or 95^th^-quantile of the SNP-wise ratios formed by the *m*_*i*_*m*_*j *_SNP pairs. Significant *Q*_*ij *_values were identified by the *curve *method.

6. An alternative way to evaluate the statistical significance is the *Rank *method. Similar to Step 4, we have 10 × *l *× (*l*-1)/2 points in each bin. The values of *R *in each bin are ranked from 1 to 10 × *l *× (*l*-1)/2. For each gene pair, the observed *R *value received such a rank value *T*, and the *R *value from permutation *p *received a rank value *T*^(*p*)^. The *p*-value of the gene-pair is then the proportion of *T*^(*p*) ^≥ *T*. In this study a significance level of 0.01 is used. The same procedure can also be applied to the *Q *statistics calculated in Step 5.

## Results

**Figure 1:**
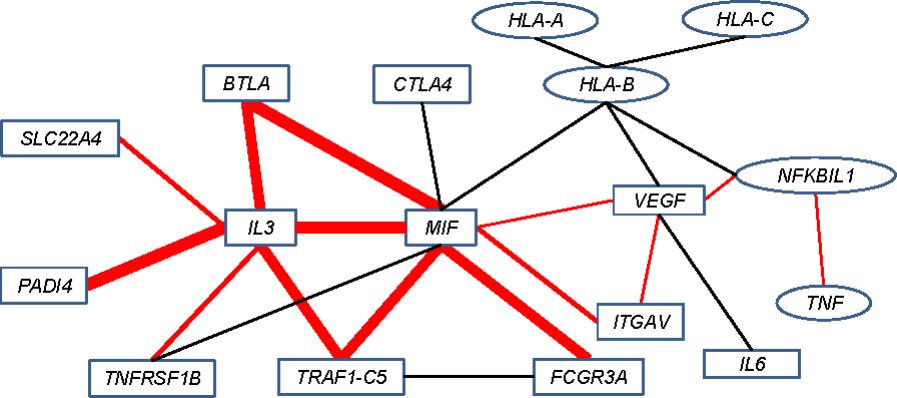
**Association network with significant interactions identified**. All significant interactions identified for Gene Set II are plotted. Interactions in red were also identified in Gene Set I. Different line widths indicate interactions identified by different numbers of proposed procedures.

From the average maximum marginal statistics, we observed that all *HLADRB1*-related gene pairs have the strongest *M *values. We applied both *Mean-ratio *and *Quantile-ratio *methods to capture the interactions. Thirteen interactions are significant at (*p *< 0.01) by the *Rank *method. Seven interactions were identified by the quantile method at 90^th ^and 95^th ^level. One interaction (*ITGAV *and *VEGF*) was identified by the 90^th^-*Quantile Rank *method. The *Curve *method identified 10 interactions, all of which were overlapping with that of the *Rank *method. Table 2 shows the detailed selection results. In Figure 1, we reported the 14 significant gene-gene interactions from Gene Set I (3.51 significant interactions expected by chance) using red lines.

**Table 2:** Selected interactions by different proposed procedures (*p*-value < 0.01)

Curve method			Rank method		
	
Mean Ratio	90% Quantile	95% Quantile	Mean Ratio	90% Quantile	95% Quantile
*IL3 BTLA*	*IL3 BTLA*	*IL3 BTLA*	*IL3 BTLA*	*IL3 MIF *^a^	*IL3 BTLA*
*IL3 MIF*	*IL3 MIF*	*IL3 MIF*	*IL3 MIF*	*IL3 PADI4*	*IL3 MIF*
*IL3 PADI4*	*IL3 PADI4*	*IL3 PADI4*	*IL3 PADI4*	*IL3 TRAF1-C5*^a^	*IL3 PADI4*
*IL3 SLC22A4*	*IL3 TRAF1-C5*	*IL3 TRAF1-C5*	*IL3 SLC22A4*	*ITGAV VEGF*	*MIF BTLA*
*IL3 TNFRSF1B*	*MIF BTLA*	*MIF BTLA*	*IL3 TNFRSF1B*	*MIF BTLA*^a^	*MIF FCGR3A*
*IL3 TRAF1-C5*	*MIF FCGR3A*		*IL3 TRAF1-C5*	*MIF FCGR3A*^a^	*MIF TRAF1-C5*
*MIF BTLA*	*MIF TRAF1-C5*		*MIF BTLA*	*MIF TRAF1-C5*^a^	
*MIF FCGR3A*	*FCGR3A*		*MIF FCGR3A*		
*MIF ITGAV*	*TRAF1-C5*^a^		*MIF HLA-B*^a^		
*MIF TRAF1-C5*			*MIF ITGAV*		
*MIF CTLA4*^a^			*MIF TNFRSF1B*^a^		
			*MIF TRAF1-C5*		
			*MIF VEGF*		
			*NFKBIL1 HLA-B*^a^		
			*NFKBIL1 VEGF*		
			*NFKBIL1 TNF*		
			*VEGF IL6*^a^		
			*VEGF HLA-B*^a^		
			*HLA-A HLA-B*^a^		
			*HLA-B HLA-C*^a^		

^a ^Significant interactions only identified in Gene Set II

For Gene Set II (2.76 significant interactions expected by chance), in addition to the interactions identified for Gene Set I (Table 2), another seven interactions were found to be significant by the *Mean-ratio Rank *method and two additional interactions were found significant by the *Curve *method. Figure 1 displays the extended networks with these interactions. As seen in Figure 1, *MIF *seems to be a "hub" in the association network with nine significant interactions. In addition, *IL3*, *VEGF*, and *HLA-B *also have at least five significant interactions.

Morand et al. [14] suggested that *MIF *is a pivotal mediator of RA. In addition, it has been implicated in many roles such as leukocyte recruitment, activation, and the production of pro-inflammatory cytokines. All of the roles contributed to the pathology of RA and showed the importance of MIF. Many interactions identified in our study have been previously discussed in the literature. It has been demonstrated that serum and synovial fluid levels of *MIF *were well correlated with the *VEGF *levels in patients with RA [15]. Nakahara et al. [16] indicated that *IL-6 *blockade directly suppressed *VEGF *production in synovial fibroblasts and may consequently reduce serum *VEGF *levels in patients with RA. Kiriakidis et al. [17] also showed that *VEGF *production in human macrophages was NF-*κ*B-dependent, which agreed with our finding of interaction between *VEGF *and *NFKBIL1*. *VEGF *may be directly involved in the activation of RA monocytes and synoviocytes, producing *TNF *and *IL-6 *via a receptor-coupling event [18]. As noted in Yoo et al. [18], this may imply a direct interaction between *VEGF *and *IL-6 *or an indirect interaction between *VEGF *and *TNF *via *NFKBIL1*, also found in our results. Onodera et al. [19] demonstrated that *MIF *enhanced NFKB binding activities of the nuclear extracts from RA synovial fibroblasts. The *NFKBIL1*, a divergent member of I-*κ*-B proteins that is an inhibitor for NFKB complex, may have potential interaction between *MIF *and *NFKBIL1*. In Figure 1, we showed that the indirect connection between *MIF *and *NFKBIL1 *via *VEGF*. Furthermore, the relation between *TNF *and NFKB-dependent signaling pathway of RA patients was discussed by Youn et al. [20].

## Conclusion

In this paper, many relevant candidate regions reported in the literature were chosen for a more detailed analysis. We applied a recently developed method by Lo et al. [13] to identify potential gene-gene interactions that are associated with the susceptibility of RA. First, we found a strong marginal signal between *HLADRB1 *and RA. In addition, the *Rank *and the *Curve *methods based on 1000 permutations identified a number of gene-pairs that are significantly associated with RA, many of which were previously reported in the literature. The association network constructed in our paper may offer additional evidence and insight on gene-gene interactions in the development of RA. Additional experiments and independent data are required to confirm our findings.

## List of abbreviations used

CGEMS: Cancer Genetic Markers of Susceptibility; GAW16: Genetic Analysis Workshop 16; GTD: Genotype-trait distortion; NARAC: North American Rheumatoid Arthritis Consortium; RA: Rheumatoid arthritis; SNP: Single-nucleotide polymorphism

## Competing interests

The authors declare that they have no competing interests.

## Authors' contributions

SL and TZ conceived and designed the research. C-HH, BQ, and LC carried out the data analysis and computation. C-HH, JX, S-HL, and TZ analyzed the results and prepared the manuscript. All authors read and approved the final manuscript.
